# Typical Diagnostic Reference Levels of Common Indications for Computed Tomography Scans Among Adult Patients in Uganda: a Cross-sectional Study

**DOI:** 10.21203/rs.3.rs-2683913/v1

**Published:** 2023-03-22

**Authors:** Festo Kiragga, Geoffrey Erem, Harriet Kisembo, John Mark Kasumba Mayanja, Aloysius G. Mubuuke, Ethel Nankya, Kevina Nalwoga

**Affiliations:** Gulu University; Makerere University; Mulago National Referral Hospital; Mulago National Referral Hospital; Makerere University; Boston University; Makerere University

**Keywords:** DRL, Indication Based-Diagnostic Reference Level, Computed Tomography scan, Adult, Uganda

## Abstract

**Background:**

Medical exposure to ionizing radiation has increased due to an increase in the number of computerized tomography (CT) scan examinations performed. The International Commission on Radiological Protection (ICRP) recommends indication-based diagnostic reference levels (IB-DRLs) as an effective tool that aids in optimizing CT scan radiation doses. In many low-income settings, there is a lack of IB-DRLs to support optimization of radiation doses.

**Objective:**

To establish typical DRLs for common CT scan indications among adult patients in Kampala, Uganda.

**Methodology:**

A cross sectional study design was employed involving 337 participants enrolled from three hospitals using systematic sampling. The participants were adults who had been referred for a CT scan. The typical DRL of each indication was determined as the median value of the pooled distribution of CTDIvol (mGy) data and the median value of the pooled distribution of total DLP (tDLP)(mGy.cm) data from three hospitals. Comparison was made to anatomical, and indication based DRLs from other studies.

**Results:**

54.3% of the participants were male. The following were typical DRLs for: acute stroke (30.17mGy and 653mGy.cm); head trauma (32.04mGy and 878mGy.cm); interstitial lung diseases/ high resolution chest CT scan (4.66mGy and 161mGy.cm); pulmonary embolism (5.03mGy and 273mGy.cm); abdominopelvic lesion (6.93mGy and 838mGy.cm) and urinary calculi (7.61mGy and 975mGy.cm). Indication based total Dose Length Product (tDLP) DRLs was lower than tDLP DRLs of a whole anatomical region by 36.4% on average. Most of the developed typical IB-DLP DRLs were lower or comparable to values from studies in Ghana and Egypt in all indications besides urinary calculi while they were higher than values in a French study in all indications besides acute stroke and head trauma.

**Conclusion:**

Typical IB-DRLs is a good clinical practice tool for optimization of CT doses and therefore recommended for use to manage CT radiation dose. The developed IB-DRLs varied from international values due to differences in selection of CT scan parameters and standardization of CT imaging protocols may narrow the variation. This study can serve as baseline for establishment of national indication-based CT DRLs in Uganda.

## Background

The technological advancement in Computerized Tomography (CT) has increased its clinical applications with multidetector CT (MDCT) scans worldwide (1, 2). CT scanners contribute the highest (43%) collective effective dose of radiation to the population among all medical imaging modalities(1). This increases the long-term risk for some cancers by 2% (3). The International Commission on Radiological Protection (ICRP) among other international bodies has issued recent calls for development and utilization of CT indication based-DRLs (IB-DRLs) for better optimization of CT doses per examination within or among CT facilities in preference to CT anatomical based DRLs(4–8). This added onto the Bonn Call for action by IAEA and WHO to strengthen radiation dose optimization through DRL establishment and use within a decade from 2017(9). The IB-DRL is superior to a single anatomical DRL that cannot logically optimize doses for all the different indications with different image quality requirements in a body region as indicated by ICRP, IAEA and European Society of Radiology (4–6, 8). However, IB-DRLs are yet to be considered a priority by the Atomic Energy Councils in low Middle-Income Countries (LMICs) as evidenced by limited published data on IB-DRLs from some studies conducted in Ghana, Egypt and Nigeria (10–12). This current study was conducted in Uganda, a low-resource setting, but with increasing use of CT in patient care due to the availability, relative affordability, high resolution images and fast image acquisition of CT equipment (13). Currently, in many LMICs, there is limited published data on DRLs (14). Some of such studies that have been conducted to establish anatomical CT DRLs in adults and pediatrics (15–19). However, CT IB-DRLs are lacking at national level and for smaller groups of hospitals in Uganda. DRLs are recommended as one of the tools that can be employed to minimize ionizing radiation used in medical exposures to as low as reasonably achievable levels (ALARA) (6). Therefore, this study sought to establish typical IB-DRLs for common CT scan indications among adult patients in Kampala, Uganda to aid CT dose optimization since national indication based-DRLs (IB-DRLs) are lacking. Findings from the study can be used by many other LMIC settings to establish their own IB-DRLs.

## Methods And Materials

### Study design

A cross-sectional design was used.

#### Selection of the participating CT facilities

At the time of the study, the central region of Uganda where Kampala is located, had 13 out of the 25 CT scanners in the country. Since the study’s target was to establish DRLs using less than 10 CT scanner rooms/hospitals, according to the available financial and time resources, the type of DRL it was to develop was a typical DRL as defined in ICRP publication 135(6). For ethical reasons the hospitals were anonymized and coded with alphabetical letters. Five tertiary hospitals with a high bed capacity, wide range of radiological services and functional CT scanners within Kampala were selected randomly to represent the public sector (Hospital C), private not for profit sector (Hospital A) and private for-profit sector (Hospitals B, D and E). A survey was done at the selected hospitals to ascertain the monthly number of adult head, chest and abdominopelvic CT scan examinations performed and their clinical indications with the results presented in Table 1. Hospitals with the highest number of head CT examinations were A, B and C. Hospitals with the highest number of chest CT examinations were A, D and E. Hospitals that performed the highest number adult CT examinations in most body regions were selected as the study sites and these included Hospitals A, B and C. Permission was sought from the administrations of the three study sites, each of which had one CT scanner.

#### Selection of the most common adult CT scan indications

The two indications most frequently examined for among head CT scans, chest CT scans and abdominopelvic CT scans at each of the five hospitals were identified and are presented in Table 1. The indications that were most frequently examined for across all the five hospitals or across most of the hospitals were selected as the most common indications for adult CT scans and these included head trauma and acute stroke among head CT scans, pulmonary embolism (PE) and interstitial lung diseases/high resolution chest CT scan (ILD/HRCT) among chest CT scans and abdominopelvic lesion (ABDPL) and urinary calculi (UC) among abdominopelvic CT scans.

### Inclusion and exclusion criteria

Patients who had been referred to the hospital CT units for examination were screened to check for eligibility which included: age of 18 years and above (adult); a common CT scan indication, informed consent; weight of 50–90kg for those with the indication of either interstitial lung disease, pulmonary embolism, abdominopelvic lesion or urinary calculi as the size of the chest and abdomen varies with body weight; and any body weight for head trauma and acute stroke as the size of the head does not vary substantially with change in weight (6). CT examinations with incomplete raw data on the CT console and those with mixed indications (e.g., stroke or brain mass) were excluded.

Since data was not collected from large electronic data bases, and was collected for a few participants using paper forms, it was important to restrict the weight range to minimize variation in the CT doses of indications within the chest and abdomen regions whose size varies with body weight. Therefore DRLs of PE, ILD/HRCT, ABDPL and UC were developed for a standardized adult of 50–90 kg as suggested by ICRP (6).

### Sampling and sample size estimation

Twenty participants were recruited for each CT indication per hospital as recommended by ICRP besides for urinary calculi that received few participants during the study duration (6). A total sample size of 337 participants were recruited in this study. Participants were recruited by systematic sampling for each indication at each hospital according to the number of CT examinations performed at a particular hospital for a specific indication.

### Study procedure and data collection

#### Quality assurance tests performed on the CT scanners

Prior to data collection, quality control (QC) tests were performed on all the CT scanners to verify the radiation doses displayed on the consoles. These tests included radiation surveys using a Geiger Muller Counter to ascertain the safety of the CT bunkers; image quality performance tests done using the CIRS Model 610–05 AAPM CT Performance Phantom and they included CT Number uniformity, CT image noise, CT number accuracy and linearity, spatial resolution and scale tests; CT dose delivery accuracy test done using the 16 cm and 32 cm diameter polymethylmethacrylate (PMMA) CTDI phantoms plus the RaySafe CT dosimetry kit to measure the actual radiation dose output from the CT scanner (CTDIvol and DLP).

There was a discrepancy between the actual CT dose emitted by the CT scanner and the CT dose displayed on the console as shown below.

**Table:** 

CT scanner	Hospital A	Hospital B	Hospital C
CTDI (%) Difference between actual CT scanner dose output and value displayed on CT console	Head CT-20.68%	Head CT-63.89%	Head CT-41.03%%
	Abdomen CT-10.58%	Abdomen CT-75.99%	Abdomen CT-65.64%
	Chest CT-15.63%	Chest CT-69.94%	Chest CT-53.33%

There was need to correct the CT doses displayed on the consoles into the actual emitted doses. The actual radiation dose output from the CT scanners was recorded as X and the dose displayed on the CT console was recorded as Y for head CT examinations and abdomen CT examinations. A formula for calculating the actual radiation dose emitted by the CT scanner during an examination was then developed as (Y)/conversion factor. The conversion factor being (Y)/(X). The conversion factors for head CT examinations and abdomen CT examinations were interpolated for each CT scanner to get the specific conversion factor for chest CT examinations.

These conversion factors were used on the collected displayed CT doses as there were no in-country Siemens biomedical engineers at the time of the study to go on with CT machine calibration using the conversion factors. The IAEA recommends use of conversion factors to verify the displayed CT radiation doses (4).

#### Data collection

Among the patients who had been referred for a CT examination at the hospital CT unit, those who met the study’s eligibility criteria by age and CT indication were approached to obtain informed consent. The consented participants with the indications of acute stroke and head trauma were enrolled. The consented participants with the indications of PE, ILD/HRCT, ABDPL and UC were weighed and those within 50–90 kg were enrolled into the study. The age, gender, weight and CT scan indication of the participants were recorded. After the CT examination had been performed by a radiographer, the CT scan parameters (which included mAs, kVp, pitch, rotation time (Trot), slice thickness, scan length, number of scan sequences and contrast use) and the radiation dose output from the CT scanners (CTDIvol and DLP) were recorded from the CT console. The quality of all CT images of the recruited participants was assessed by a radiologist at each hospital, using a scale adopted from the IAEA CT dose data collection tool, as acceptable, higher than needed or unacceptable and was recorded too. All data was recorded onto a paper form using a CT data collection tool adapted from the international atomic energy agency (IAEA).

### Study variables

The study variables were kVp, total mAs, effective mAs, slice thickness, scan length, contrast use, number or range of scan sequences, total DLP, CTDIvol.

Image quality was assessed using a 3-point scale adapted from the International Atomic Energy Agency (IAEA) CT dose data collection tool. Every image was scored for overall quality by the radiologist on a 3-point scale which included: 1 = Acceptable, 2 = Higher than needed and 3 = Unacceptable.

### Data management and analysis

Data was de-identified, checked daily for completeness and kept under lock and key. It was entered into excel for cleaning, error checks, editing and storage. Using excel, the recorded CT dose (Y) was converted into the actual radiation dose emitted by the CT scanner during examination of a participant using the formula; actual radiation dose emitted (A) = (Y)/conversion factor.

The actual CT doses and other data were then exported into and analyzed using R and R studio version 4.10. The Shapiro test was used to test for normality of the data and the data was found to be skewed. Descriptive analysis of the data was performed to include frequencies, the median and interquartile range.

The typical IB-DRLs were determined following the method recommended in ICRP publication 135 (6). The typical DRL for each indication was determined as the median value of the average CTDIvol of the whole examination and the median value of the total DLP using dose data of 60 participants combined from all three hospitals, for all indications besides urinary calculi. For urinary calculi, the typical DRL was developed using data from a total of only 30 participants: 20 from Hospital B and 10 from Hospital A. Only 7 participants were recruited at Hospital C during the study period, therefore these were very few to be included in the calculation of the typical DRL for urinary calculi as the minimum number of participants that can be included in the calculation of a DRL for most examinations generally maybe 10 (4, 6).

In addition, an overall IB-DRL for each indication was calculated as the 75th percentile of the median CTDIvol values and the median total DLP values from the three hospitals for comparison to national DRLs from other studies including anatomical based national DRLs in Uganda where the study was conducted from and IB-DRLs from international studies in Ghana, Egypt and Ghana (10, 11, 17, 20).

## Results

The purpose of this study was to establish typical DRLs for common indications of CT scans among adult patients.

### Characteristics of the sample population

There were 154 (45.74%) females. The overall median age was 53.5(36.25–67.75) years with the youngest participants being those with head trauma at 35(28–45) years and the eldest being those with stroke at 57.5(44.5–70) years. The overall median weight for all indications in the study excluding head trauma and stroke was 74(63.6–79.7) kg. The distribution of the study sample by age, weight, and gender in each CT indication at all hospitals combined is presented in Table 2.

### CT scanner characteristics

All the study hospitals possessed CT scanners manufactured by Siemens with that of Hospital C being a 16-slice scanner and the other characteristics of the CT scanners are presented in Table 3.

### Scanning parameters per CT indication

All CT dose data for each indication was collected from images of acceptable quality as subjectively assessed by radiologist. The median x-ray tube kilovoltage of 130 kVp was used to acquire CT images for each indication unlike for pulmonary embolism (PE) in which 110 (110– 114.25) kVp was used. Contrast material was only used in the indications of PE, ABDPL and UC. The rest of the scan parameters that were used to perform the CT examinations for each indication in the study are presented in Table 4.

### Typical IB-DRLs

The typical CTDIvol DRLs of indications within the head, chest and abdomen were comparable with a varifying factor (vf) of 1.1. The typical DLP DRL of head trauma was 1.34-fold higher than for acute stroke. The typical DLP DRL for PE was 1.7-fold higher than for ILD/HRCT. The typical DLP DRL for UC was higher to that of ABDPL by a vf of 1.2-fold. The developed typical DRLs at 50th percentile plus the overall DRLs at the 75th percentile are presented in Table 5.

#### Comparison of the developed overall DRLs at 75th percentile (P) to published anatomical based national DRLs (AB-NDRLs) in Uganda.

The CTDIvol DRLs of all indications were lower than the AB-CTDIvol DRLs of the corresponding anatomical regions. A comparison of the current study’s DRLs for the various indications to the Ugandan AB-NDRLs of the corresponding anatomical regions revealed that the DLP DRLs were only lower than the AB-national DLP values in the indications of head trauma (lower by 27.4%), acute stroke (lower by 42.4%), and ILD/HRCT (lower by 39.4%) (17) as presented in Table 6.

#### Comparison of the overall IB- DRLs at 75th percentile in the current study to some of the published national IB-DRLs at 75th percentile.

**Figure 1:**
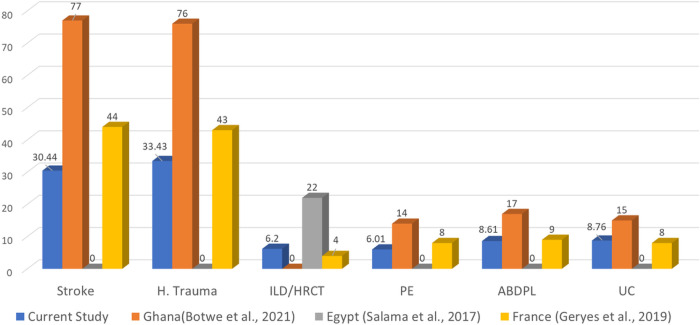
Comparison of the current study’s overall IB-CTDIvol DRLs at 75th P to published national IB-CTDIvol DRLs at 75th P

The CTDIvol DRLs were lower than Ghana’s (10) in all indications (acute stroke, head trauma, PE, ABDPL and UC) and lower than Egypt’s (11) in ILD/HRCT as presented in Fig. 1. The CTDIvol DRLs were lower than France’s (20) in acute stroke and head trauma, comparable to France’s (20) in ABDPL and UC and only higher than France’s (20) in ILD/HRCT as presented in Fig. 1.

**Figure 2:**
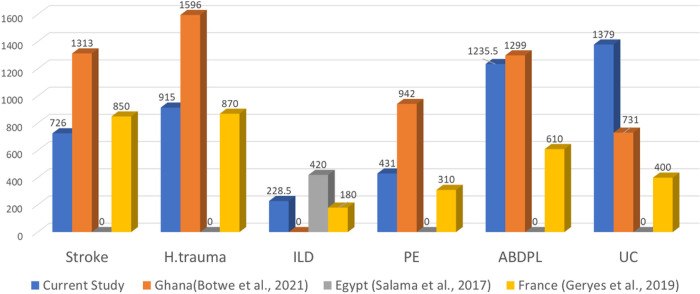
Comparison of the current study’s overall IB-DLP DRLs at 75th P to published national IB- DLP DRLs at 75th P

The DLP DRLs were mostly lower than those of Ghana (10) in the indications of acute stroke, head trauma and PE, but higher than Ghana’s (10) only in UC and only comparable to Ghana’s (10) in ABDPL as presented in Fig. 2. The DLP DRL of ILD/HRCT was also lower than Egypt’s (11) as presented in Fig. 2. The current study’s DLP DRLs were mostly higher than those of France (20) in the indications ILD/HRCT, PE, ABDPL and UC and were only lower than France’s (20) values in the indications of acute stroke and head trauma as presented in Fig. 2.

## Discussion

The purpose of this study was to establish typical DRLs for common CT indications among adult patients in Uganda. In this study, IB-DRLs for common indications of CT examinations among adults were developed as typical DRLs set at the 50^th^ percentile of the pooled distribution of dose data from 12% (3/25) of the CT scanners in Uganda, a low-income country. The common CT scan indications were similar to those in a study within Ghana and in Europe (10, 21).

The overall median weight for all indications excluding head trauma and stroke was 74(63.6–79.7) kg with pulmonary embolism having the most heavy patients with 78(71.5–85) kg and interstitial lung diseases having the least heavy patients with 69.5(60–77) kg. This overall median weight was within that of a standard adult population defined by ICRP with 70+/−20kg. These findings were also similar to those of other studies in which heavier participants suffered more recurrent episodes of venous thromboembolism and pulmonary embolism (22) and lower weight was associated with disease progression in interstitial lung diseases (23).

### CT scanning parameters:

#### X-ray tube voltage (measured in peak kilovoltage, kVp):

The tube voltage of 130kVp that was used for all indications besides pulmonary embolism was higher than values used in other studies for example higher than 118 (±8.3) to 121.8(±7.4) in Ghana (10), 120 kVp in Egypt (11) and (100 to 120) kVp in France (20). The tube voltage of 110 (110– 114.25) kVp for pulmonary embolism was similar to trends in some studies for example (100–120)kVp in France (20) while this kVp for PE was lower than values in other studies for example 117.8(±4.0) in Ghana. There is potential to reduce CT radiation doses by lowering kVp for patients of smaller body weight, especially for ILD/HRCT by setting a specific kVp for a particular weight category on the CT machines at the hospitals as kVp in the recent CT machine models is fixed for an examination and is not modulated automatically during an examination to suit body size like it happens for x-ray tube current (24). There is hope though that newer CT scanner models may bring further improvements in the range of available tube voltages and more advanced automatic tube voltage selection tools which can automatically alter kVp according to patient size to achieve ALARA doses (25).

#### X-ray tube current-time product (measured in milliampere per second, mAs):

Indications that did not use contrast material like ILD/HRCT generally had a lower effective mAs and a lower total mAs and compared to those that used contrast material like ABDPL, because of the lower attenuation in the absence of iodinated contrast material in the noncontrasted examinations. Indications within the chest generally had a lower total mAs than those in the abdomen and head because of the lower density/attenuation of the chest tissues for which tube current modulation software automatically reduces the mAs during the scan to minimize radiation dose (24). This trend was similar to the trend in the mAs used in a study within Ghana (10).

#### Scan length:

The scan length for head trauma was longer than that for acute stroke due to the inclusion of a longer part of the neck to rule out concomitant cervical spine injury for early management to mitigate complications similar to a studies in Ghana and Uganda (10, 15, 16, 19).

The scan length for PE was shorter than that for ILD/HRCT due to the exclusion of the most peripheral chest parts in which emboli are not generally seen within the terminal pulmonary arterioles. This trend was similar to that in a study within Ghana (10). This trend of scan length was largely within the recommended limits of the anatomical extent for PE examinations which extends from the aortic arch to the base of the hear (26).

The scan length for UC was shorter than that for ABDPL probably due the focus on calculi within the upper urinary system as per the scan request form with less inclusion of the most distal parts of the pelvis after the urinary bladder This was dissimilar to findings in a study within Ghana (10) in which both ABDPL and kidney stones had similar scan lengths. This was also dissimilar to the recommendation by ESR that advises the scan length for UC to extend longer than that of ABDPL (appendicitis) i.e., from inferior margin of T10 to lower edge urinary bladder (approximately at lower edge of pubis symphysis) for UC and from inferior margin of T10 to superior border of pubis symphysis (26).

Scan length depends on the height of the participant and the extent of the anatomy that has to be demonstrated therefore it is important to keep it within limits that answer the clinical question for the CT scan.

#### Number of scan sequences:

Only a precontrast scan sequence was used to examine head trauma, acute stroke and ILD/HRCT as the tissues provide adequate natural contrast to allow visualization of the questioned pathology in these indications similar to a study within Ghana (10).

In the chest, the scan sequences for PE were more than for ILD/HRCT partly due to the higher image quality requirements with contrast use and partly due to the inclusion extra sequences at some hospitals for example a precontrast HRCT phase to rule COVID-19 pneumonia during the pandemic and a delayed phase to rule emboli in pulmonary veins. These findings differed from other studies in Ghana and France that used less sequences (1–2) (10, 20).

In the abdomen, a higher-than-expected number of scan sequences were used for UC contrary to a single noncontrast sequence which is usually adequate as calculi provide high contrast to visualize them easily (4, 27). Many postcontrast scan sequences were used in UC and ABPL mainly due to absence of standardized scan protocols optimized for these indications and these findings differed from other studies that used one noncontrast scan sequence for UC and one postcontrast scan sequence for ABDPL in Ghana, France and Europe (10, 20, 21).

There is potential to reduce CT doses by lowering the number of scan sequences for PE, ABDPL and UC through adherence to the developed indication-based examination protocols where they exist or development of the optimized indication-based scan protocols where they are absent.

#### Slice thickness:

The acquisition slice thickness was similar for all indications across all body regions even for pulmonary embolism and HRCT/ILD which require thinner slice acquisition due to the need for higher image quality. The slice thickness for indications in the head(head trauma and acute stroke) and for abdominopelvic indications (ABPL and UC) was expectedly comparable as the indications in both regions do not necessarily require very thin slice thickness to have diagnostic images similar to findings in a study within Ghana (10). The slice thickness finding for PE differed from findings of a study within Ghana in which the slice thickness for PE was lower than that of other indications in the chest region (10). It is important to note that image quality in the current study was assessed subjectively and was adequate for PE similar to the high image quality in the cited study (10) that assessed the quality objectively. There is need to assess image quality objectively in future studies to better assess if a slice thickness greater than 1mm provides good enough image quality for PE assessment.

### The typical DRLs

The CTDIvol DRLs were observed to be comparable for different indications within the same body regions similar to a study in Ghana (10). The CTDIvol DRLs in the head region (acute stroke and head trauma) and abdomen region (abdominopelvic lesion and urinary calculi) were similar due to use of similar CT scan parameters (effective mAs). The CTDIvol DRLs in the indications within the chest region (ILD and PE) ended up being comparable due to use of a combination of CT scan parameters that raise and reduce CTDIvol i.e., in ILD, the lower pitch raises the CTDIvol while the lower effective mAs reduces the CTDIvol while in PE, the higher effective mAs raises the CTDIvol while the higher pitch reduces the CTDIvol.

The DLP DRLs were observed to differ among indications with the same body region due to the differences in image quality needs and use of different scan parameters. The DLP DRL for head trauma being higher than for acute stroke can be explained by a longer scan length due to the need to rule out cervical spine injury similar to a study within Ghana (10). The DLP DRL for PE being higher than for ILD/HRCT can be explained by the higher image quality need that requires contrast use and therefore a higher total mAs, plus the use of a higher-than-expected number of scan sequences. This trend of CT doses within the chest findings were similar those in a study within France (20). The DLP DRL for UC being unexpectedly higher to that for ABDPL can be explained by the absence or less frequent utilization of an optimized scan protocol for UC which allowed room for use of postcontrast sequences which were high in number than those for ABDPL. This finding was different from that in other studies in which the DLP DRL for UC was lower than for ABDPL due to use of a single noncontrast scan sequence for UC in an optimized protocol (10, 11, 20).

### Comparison of the IB-DRLs to anatomical based national DRLs (AB-NDRLs) in Uganda.

The CTDIvol DRLs of all indications being lower than corresponding anatomical based values was probably due to use of a lower total mAs. The IB-DLP DRLs for head trauma, acute stroke and ILD/HRCT being lower than AB-DLP DRLs for head CT and chest CT scans respectively can be explained mainly using a lower total mAs. The need for lower image quality requirements without need for contrast media may have further contributed to the DLP DRLs of acute stroke, head trauma and ILD/HRCT being lower. Some of the current study’s indication based DLP DRLs were lower than DRLs of an anatomical region by 36.4% on average similar to findings in a studies within Finland (28) and Switzerland (29) that found IB-DRLs lower by 20% and by 21–32% respectively.

However, the DLP DRL for PE was higher than for chest CT scans due to use of thinner acquisition slice thickness and generally a higher need for higher image quality similar to another study in Switzerland (29).

The DLP DRL for ABDPL in the current study ended up being comparable to the DLP value for abdomen CT scans (Erem et al., 2022) due to a combination of CT scan parameters that raise the DLP including a longer scan length of 49.12 (46.29–53.09)cm and a thinner slice thickness of 3 (1–5) mm in ABDPL examinations compared to a scan length of 41.25 (32.0–63.3) cm and to a slice thickness of 3.75mm slice thickness that were used in abdomen CT scans (Erem et al., 2022), and due to use of a lower total mAs of 4783 (3496–7088) mAs which reduces the DLP compared to 6115.5 (3619–9869) mAs of abdomen CT scans (Erem et al., 2022). The finding of the DLP DRL of an ABDPL being comparable to the DLP of abdomen CT scans (17) differed from findings in a study within Switzerland(29) that found the DLP DRL for appendicitis to be lower than for abdomen CT scans because this cited study in Switzerland used optimized protocols for appendicitis with probably fewer scan sequences.

The DLP DRL for UC in the current study ended up being unexpectedly comparable to the high DLP value for abdomen CT scans (17) due to a combination of CT scan parameters that raise the DLP including many scan sequences that included many postcontrast phases (0–6), a longer scan length of 46.88 (42.83–49.13) cm and a thinner slice thickness of 3 (1–5) mm in UC examinations compared to 41.25 (32.0–63.3) cm scan length and to 3.75mm slice thickness that were used in abdomen CT scans (17), and due to use of a lower total mAs of 4888 (3324–6221) mAs which reduces the DLP compared to 6115.5 (3619–9869) mAs of abdomen CT scans (17). The finding of the DLP DRL of UC being comparable to the DLP of abdomen CT scans (17) differed from findings in a study within Switzerland(29) that found the DLP DRL for kidney stones to be lower than for abdomen CT scans because this cited study in Switzerland used optimized protocols for kidney stones with probably fewer scan sequences.

### Comparison of the overall IB- DRLs at 75th percentile to some of the published national IB-DRLs at 75th percentile

#### Acute Stroke

The CTDIvol and DLP DRLs of acute stroke being much lower than values in Ghana (10)was mainly due to use of a lower effective mAs of 169-(160–203) compared to the higher tube loading of 238.0 (± 80) mAs in Ghana(10). The reasons for the CTDIvol and DLP DRLs for acute stroke being lower than France’s values (20) were not ascertained because very few CT scan parameters were mentioned in the cited study within France which limited more comparative analysis.

#### Head Trauma

The CTDIvol and DLP DRLs of head trauma being much lower than values in Ghana (10) was mainly due to use of a lower effective mAs of 181(168–206) compared to the mAs used in Ghana (229.4 ± 73.5 mAs) (10). The reasons for the CTDIvol DRL for head trauma being lower than, and the DLP DRL for head trauma being comparable to, the corresponding values in France(20) were not ascertained as very few scan parameters were mentioned in the cited study in France for more comparative analysis.

#### ILD/HRCT

The CTDIvol and DLP DRLs of ILD/HRCT being much lower than the values in Egypt (11) was probably due to use of a lower effective mAs of 61.5 compared to the mAs used in Egypt within a range of (100 minimum mAs to 430 maximum mAs)(11).

The CTDIvol and DLP DRLs for ILD/HRCT being higher than values in France (20) was due to use of a higher kilovoltage of 130 compared to 100 kVp used in most examinations (61 %) the cited study within France(20).

#### PE

The CTDIvol and DLP DRLs of PE being lower than values in Ghana (10) was probably due to use of a wider slice thickness of 3 (1–5) mm compared to Ghana’s (2.20 ± 1.7)mm, a lower peak tube kilovoltage of 110 (110– 114.25) kVp compared to Ghana’s (117.8 ± 4.0)kVp, and a lower effective mAs of 98 compared to Ghana’s tube loading of (167.5 ± 92.9) mAs (10). Even though the current study’s DRLs for PE were lower than Ghana’s, the quality of PE examination images was of acceptable quality to make a diagnosis as assessed by radiologists.

The reason for the CTDIvol DRL of PE being lower than the value in France (20) was unknown as the cited study did not mention its scan parameters which limited comparative analysis.

The corresponding DLP DRL for PE was instead higher than France’s value (20) due to use of a higher tube peak kilovoltage of 110 (110–114.25) compared to 100kVp used in most examinations (55%) in the French study (20), probably due to frequent use of unoptimized examination protocols with a higher number of scan sequences (1–4) and probably due to a longer scan length compared to the usually optimized PE protocols in European countries(26).

#### ABDPL

The CTDIvol DRL being lower than Ghana’s value (10) was due to using a lower effective mAs of 98(81.33) compared to Ghana’s tube loading mAs of (137.0 ± 91.4)(10).

In a comparison to the cited study in Ghana(10), it was observed that most of the scan parameters used in the current study to examine an ABDPL were higher than those in Ghana for example, the current study used a higher number of postcontrast scan sequences(1–3), longer scan length 49.12 (46.29–53.09)cm, higher kilovoltage(130kVp), and thinner slice thickness 3 (1–5)mm compared to Ghana’s single postcontrast scan sequence(1), 45.99(±4.3)cm scan length, 118.7(±7.5)kVp and 5.40(±2.6)mm slice thickness respectively (10). For this reason, the DLP DRL of ABDPL in the current study was expected to be much higher than Ghana’s value but was instead comparable due to use of a lower effective mAs of 98 compared to Ghana’s tube loading mAs of (137.0 ± 91.4)(10).

The reason for the CTDIvol DRL for ABDPL being comparable to France’s value (20) was not ascertained as the cited study did not mention much about its scan parameters which limited comparative analysis .

The corresponding DLP DRL for ABDPL being higher than France’s value (20) was due to use of a higher tube peak kilovoltage of 130 compared to 100/120 kVp used for most examinations (59%) in the French study (20) and probably due to frequent use of unoptimized examination protocols with a higher number of scan sequences (2–4) compared to the usually optimized protocols for ABDPL in European countries(26).

#### UC

The CTDIvol DRL of UC being lower than Ghana’s value (10) was due to use of a lower effective mAs of 105 compared to Ghana’s (138.4 ± 98.4) mAs (10).

The DLP DRL for UC being higher than Ghana’s value was probably due to use of a higher kVp (130), thinner slice thickness 3 (1–5) mm and a higher total number of (1–7) scan sequences compared to Ghana’s 118(±8.3) kVp, 5.3(±2.5) mm and only one noncontrast scan sequence in the examination respectively (10).

The reason for the CTDIvol DRL of UC being comparable to France’s value (20) was not ascertained because the French study did not mention much about their CT scan parameters which limited comparative analysis.

The DLP DRL for UC being higher than France’s value (20) was due to use of a higher tube peak kilovoltage of 130 compared to 100 kVp used in most examinations (69%) in this cited French study(20) and probably due to frequent use of unoptimized examination protocols with a higher number of scan sequences (1–7) which included (0–6) postcontrast sequences compared to the optimized UC protocol with only one noncontrasted scan sequence in the French study(20).

Like in other published studies, the overall DRLs of some indications at 75th P varied significantly from national IB-DRLs in other countries mainly due to the difference in the scan parameters chosen for use in the examinations including kilovoltage, acquisition slice thickness, effective mAs and number of scan sequences plus the use of protocols that are less optimized for indications (10, 11, 21, 30).

#### Study strengths and limitations

The strengths of this study include having performed QC tests on the CT scanners, use of actual CT scanner output radiation doses to develop the IB-DRLs following corrections of the doses and the development of DRLs using the required minimum of 20 participants per CT scanner room for most indications as recommended by ICRP.

The main study limitation was having selected three (3) out of 13 (23%) CT facilities in Kampala and central region of Uganda, the developed typical IB-DRLs may be less representative of the doses and practices used during examinations of the selected CT indications country-wide. However, findings from the study still give an indication that locally determining typical DRLs can optimize the use of CT equipment. Further studies should be conducted using more CT facilities to develop IB-DRLs for the common CT indications that are more representative of other settings.

## Conclusion

The typical IB-DRLs determined in this study were 30.17mGy and 653mGy.cm for acute stroke, 32.04mGy and 878mGy.cm for head trauma, 4.66mGy and 161mGy.cm for interstitial lung diseases/ high resolution chest CT scan, 5.03mGy and 273mGy.cm for pulmonary embolism, 6.93mGy and 838mGy.cm for abdominopelvic lesion and 7.61mGy and 975mGy.cm for urinary calculi.

The developed typical IB-DRLs are recommended for use to optimize CT radiation doses among adults. Most of the developed typical IB-DLP DRLs were lower or comparable to DRLs from studies in Ghana and Egypt while they were higher than DRLs in the French study due to differences in selection of CT scan parameters. Standardized indication-based examination protocols for the common CT indications should be developed for indications where they do not exist, and their use strengthened to minimize variation in the DRLs in comparison to international DRL values. This can also optimize the use of CT equipment especially in low resource settings where it is not yet widely available.

## Figures and Tables

**Table 1:** CT examinations performed among adult patients and their indications at five hospitals in Kampala during a survey.

	Hospital									
	A		B		C		D		E	
**Bed capacity**	361		80 plus		1,790		80 plus		109	
	No. of CT scans	Proportion of CT indication	No. of CT scans	Proportion of CT indication	No. of CT scans	Proportion of CT indication	No. of CT scans	Proportion of CT indication	No. of CT scans	Proportion of CT indication
**Total Head CT scans**	* **178**		* **138**		* **139**		**115**		**38**	
Head trauma	83	46.60%	52	^≠^ 37.70%	58	^≠^41.70%	46	^≠^40.00%	19	^≠^50.00%
Stroke	55	^≠^66.3%	28	20.3%	35	25.2%	41	^≠^35.7%	29	^≠^76.3%
Brain masses	33	^≠^ 60.00%	56	^≠^ 40.60%	37	^≠^26.60%	26	22.60%	19	50.00%
Others	7	3.90%	6	4.30%	9	6.50%	2	1.7%	0	0.00%
**Total chest CT scans**	* **122**		**114**		**73**		* **131**		* **119**	
Pulmonary embolism	19	15.6%	32	^≠^28.1%	22	30.1 %	32	^≠^24.4%	7	^≠^5.9%
HRCT/ILD	83	^≠^68.00%	72	^≠^63.20%	28	^≠^38.40%	94	^≠^71.80%	106	^≠^89.10%
Chest masses	20	^≠^16.40%	10	8.80%	23	^≠^31.50%	5	3.80%	6	5.00%
**Total abdominopelvic CT scans**	* **63**		* **62**		* **80**		**25**		**31**	
Abdominopelvic lesion	48	^≠^76.20%	49	^≠^79.00%	55	^≠^68.80%	17	^≠^68.00%	29	^≠^93.60%
Urinary calculi	10	^≠^15.90%	7	^≠^11.30%	9	^≠^11.30%	2	8%	0	0%
CT urogram	4	6.30%	3	4.80%	5	6.30%	3	^≠^12.0%	2	^≠^ 6.5%
Liver masses	1	1.60%	4	6.50%	8	10%	0	0%	0	0%
Abdominal CT Angiogram	0	0%	0	0%	2	2.55%	3	^≠^12%	0	0%
CT colonoscopy	0	0%	0	0%	1	1.30%	0	0%	0	0%

* High number of monthly adult CT scan examinations among the five hospitals.

≠ The top two most frequent CT scan indications examined for in a body region at a hospital.

An abdominopelvic lesion included appendicitis, pancreatitis, lymphadenopathy, mass, abscess, ascites, etc. (excluding cancer staging, liver masses).

**Table 2:** Distribution of sample population used to determine CT doses by age, weight, and gender in each indication

Indication	Median Age (years)	Median weight (kg)	Proportion of Female (%)
Acute stroke	57.5 (44.50–70)	N/A	27 (45%)
Head trauma	35 (28–45)	N/A	16 (26.67%)
ILD/HRCT	49.5 (35–71)	69.5 (60–77)	28 (47.67%)
Pulmonary embolism	48 (36.80–66)	78 (71.5–85)	43 (71.67%)
Abdominopelvic lesion	50 (37.80–65)	70.5 (63–77)	31 (51.67%)
Urinary calculi	48 (37–56)	77 (67–85)	9 (24.32%)

**Table 3:** Characteristics of the CT Scanners

CT scanner	Hospital A	Hospital B	Hospital C
CT manufacturer	Siemens	Siemens	Siemens
CT model	Somatom Perspective (DE)	Somatom Perspective (VC20B)	Somatom Go. Now
Year of installation	2013	2013	2019
Year of manufacturing	2013	2013	2018
Number of detector rows	128	128	16
Automatic exposure control	Yes	Yes	Yes
Is CTDI displayed in CTDIw or CTDIvol?	CTDIvol	CTDIvol	CTDIvol

**Table 4:** Scanning parameters used to the acquire CT images per indication

Indication	Median Total mAs	Median Effective mAs	Median slice thickness (mm)	Median Scan length (cm)	Total no. seq Mn-Mx	No. postcontrast seq
Acute stroke	1676 (1144–2852)	169 (160–203)	3 (1–5)	22.3 (20.9–26.60)	1	0
Head trauma	1930 (1393–3856)	181 (168–206)	3 (1–5)	26.80 (25.3–29.50)	1	0
ILD/HRCT	1002 (723–1202)	61.50	3(0.6–5)	34.06 (32.62–38.11)	1	0
PE	1915 (1352–2388)	98	3 (1–5)	30.77 (25.85–35.91)	1–4	1–3
ABDPL	4783 (3496–7088)	98(81.33)	3 (1–5)	49.12 (46.29–53.09)	2–4	1–3
UC	4888 (3324–6221)	105	3 (1–5)	46.88 (42.82–49.13)	1–7	0–6

For number of scan sequences, the topogram sequence and monitoring phases (e.g., in the cases of PE) were not included as it is the situation for some established DRLs. Mn-Minimum, Mx-Maximum.

**Table 5:** The typical DRLs at 50th percentile and the overall DRLs at the 75th percentile.

Indication	Typical CTDIVol (mGy)	Typical tDLP (mGy.cm)	Overall CTDIvol at 75th P (mGy)	Overall tDLP at 75th P (mGy.cm)
Acute stroke	**30.17**	**653**	30.44	726
Head trauma	**32.04**	**878**	33.43	915
ILD/HRCT	**4.66**	**161**	6.20	228.5
PE	**5.03**	**273**	6.01	431
ABDPL	**6.93**	**838**	8.61	1235.50
UC	**7.61**	**975**	8.76	1379

**Table 6:** Comparison of the developed overall IB-DRLs at 75th P with national anatomical DRLs in Uganda at 75th percentile (Units for CTDIvol are mGy and for tDLP are mGy.cm)

	ACUTE STROKE		HEAD TRAUMA		ILD/HRCT		PE		ABDPL		UC	
Study	CTDIvol	tDLP	CTDIvol	tDLP	CTDIvol	tDLP	CTDIvol	tDLP	CTDIvol	tDLP	CTDIvol	tDLP
**Current Study**	**30.44**	**726**	**33.43**	**915**	**6.20**	**228.50**	**6.01**	**431**	**8.61**	**1235.50**	**8.76**	**1379**
Uganda AB-NDRL (Erem et al., 2022)	**HEAD CT SCAN**				**CHEST CT SCAN**				**ABDOMEN CT SCAN**			
	CTDIvol		tDLP		CTDIvol		tDLP		CTDIvol		tDLP	
	56.02		1260.33		7.82		377		12.54		1418.30	

**AB-NDRL**-anatomical based national DRL.

## Data Availability

All the necessary original data and materials used in this study have been included.

## List Of Abbreviations

AEC: Atomic Energy Council
AB-DRL: Anatomical based DRL
ABDPL: Abdominopelvic lesion
ALARA: As low as reasonably achievable
CT: Computed Tomography
CTDIvol: Volumetric Computed Tomography Dose Index
DLP: Dose Length Product
DRL: Diagnostic Reference Level
HRCT: High Resolution Computed Tomography scan
IAEA: International Atomic Energy Agency
IB-DRL: Indication based DRL
ICRP: International Commission on Radiological Protection
ILD: Interstitial lung diseases
LMIC: Low Middle-Income Countries
MDCT: Multidetector Computed Tomography
PE: Pulmonary embolism
tDLP: Total Dose Length Product
UC: Urinary Calculi
vf: Varying factor
75th P: 75th Percentile
50th P: 50th Percentile
